# Protein malnutrition promotes dysregulation of molecules involved in T cell migration in the thymus of mice infected with *Leishmania infantum*

**DOI:** 10.1038/srep45991

**Published:** 2017-04-11

**Authors:** Monica Losada-Barragán, Adriana Umaña-Pérez, Sergio Cuervo-Escobar, Luiz Ricardo Berbert, Renato Porrozzi, Fernanda N. Morgado, Daniella Areas Mendes-da-Cruz, Wilson Savino, Myriam Sánchez-Gómez, Patricia Cuervo

**Affiliations:** 1 Laboratório de Pesquisas em Leishmaniose, Instituto Oswaldo Cruz, Fiocruz, Rio de Janeiro, RJ, Brasil; 2 Universidad Nacional de Colombia, Sede Bogotá, Facultad de Ciencias, Departamento de Química, Grupo de Investigación en Hormonas, Bogotá, Colombia; 3 Laboratório de Pesquisas sobre o Timo, Instituto Oswaldo Cruz, Fiocruz, Rio de Janeiro, RJ, Brasil

## Abstract

Protein malnutrition, the most deleterious cause of malnutrition in developing countries, has been considered a primary risk factor for the development of clinical visceral leishmaniasis (VL). Protein malnutrition and infection with *Leishmania infantum* leads to lymphoid tissue disorganization, including changes in cellularity and lymphocyte subpopulations in the thymus and spleen. Here we report that protein malnutrition modifies thymic chemotactic factors by diminishing the CCL5, CXCL12, IGF1, CXCL9 and CXCL10 protein levels in infected animals. Nevertheless, T cells preserve their migratory capability, as they were able to migrate *ex vivo* in response to chemotactic stimuli, indicating that malnutrition may compromise the thymic microenvironment and alter *in vivo* thymocyte migration. Decrease in chemotactic factors protein levels was accompanied by an early increase in the parasite load of the spleen. These results suggest that the precondition of malnutrition is affecting the cell-mediated immune response to *L. infantum* by altering T cell migration and interfering with the capacity of protein-deprived animals to control parasite spreading and proliferation. Our data provide evidence for a disturbance of T lymphocyte migration involving both central and peripheral T-cells, which likely contribute to the pathophysiology of VL that occurs in malnourished individuals.

Visceral leishmaniasis (VL) is a parasitic disease that causes significant morbidity and mortality worldwide^1^. In the Americas, the etiological agent of VL is *Leishmania infantum*, which is responsible for ~4,000 cases annually^1^, affecting mainly children and immunocompromised adults^2^. Although infection with *L. infantum* may be asymptomatic, malnutrition favors the progression to clinical disease^3^,^4^,^5^,^6^. Worldwide, protein malnutrition is the leading cause of immunodeficiency^7^. Protein restriction is associated with increased susceptibility to infections due to deleterious effects on the immune system, thereby compromising defense mechanisms against pathogens^8^. The role of protein malnutrition as a risk factor for the development of infectious diseases has been clearly recognized^9^,^10^.

Independently, infection or malnutrition induces similar alterations to lymphoid organs. Acute infection with intracellular parasites, such as *Trypanosoma cruzi* or *Plasmodium berghei*, yields a drastic reduction in the number of immature CD4^+^CD8^+^ double positive (DP) T cells in the thymus due to an increased apoptotic rate and abnormal egress of immature thymocytes^11^,^12^,^13^. Interestingly, similar events have been found in the thymus of malnourished animals^14^,^15^,^16^,^17^. Other factors of the thymic microenvironment, such as chemokines and extracellular matrix (ECM) components, are altered during pathological conditions such as infection or malnutrition. Increased chemokine deposition accompanied by enhanced co-localization with fibronectin has been observed in the thymus of *T. cruzi*-infected mice^18^. Malnourished children presented an enhancement of thymic ECM network containing fibronectin, laminin, and type IV collagen^19^. During experimental infection with *L. donovani*, malnutrition dysregulates the expression of chemokines and the corresponding receptors involved in the trafficking of dendritic cells and their progenitors to the lymph nodes, an event that is crucial for controlling parasite dissemination^20^,^21^.

Using a murine model of protein malnutrition and infection with *L. infantum*, we demonstrated the deleterious impact of protein restriction in infected mice, including atrophy of lymphoid organs with changes in cellularity and lymphocyte subpopulations in the thymus and spleen that ultimately favor the establishment of *L. infantum* infection. In particular, the thymus exhibited a drastic cell loss due to atrophy accompanied by a significant decrease of DP thymocytes in malnourished mice infected with *L. infantum*^22^. Based on these results, we hypothesize that defects in the mechanisms of T cell migration and/or apoptotic events could be involved in thymic atrophy and in the failure to control parasite proliferation in peripheral lymphoid organs. Here we show that protein malnutrition does affect thymocyte migration of *L. infantum*-infected animals, rather than increasing their apoptosis. Protein malnutrition modified the expression of soluble factors produced by the thymic microenvironment, with reductions of CCL5, CXCL12, IGF1, CXCL9 and CXCL10 levels in infected animals. However, T cells migrate *ex vivo* in response to chemotactic stimuli indicating that T cells preserve their migratory capability, highlighting the crucial role of the microenvironment for *in vivo* thymocyte migration. As the reduction of migratory molecules was accompanied by a higher parasite load in the spleen of malnourished mice, it is possible to suggest that a precondition of protein restriction alters cell-mediated immune responses by interfering with both central and peripheral T-cell migration and reducing the capacity of protein-deprived animals to control parasite proliferation.

## Results

### Malnourished BALB/c mice infected with *L. infantum* present decreased body weight and diminished IGF1 and leptin serum levels

**Figure 1:**
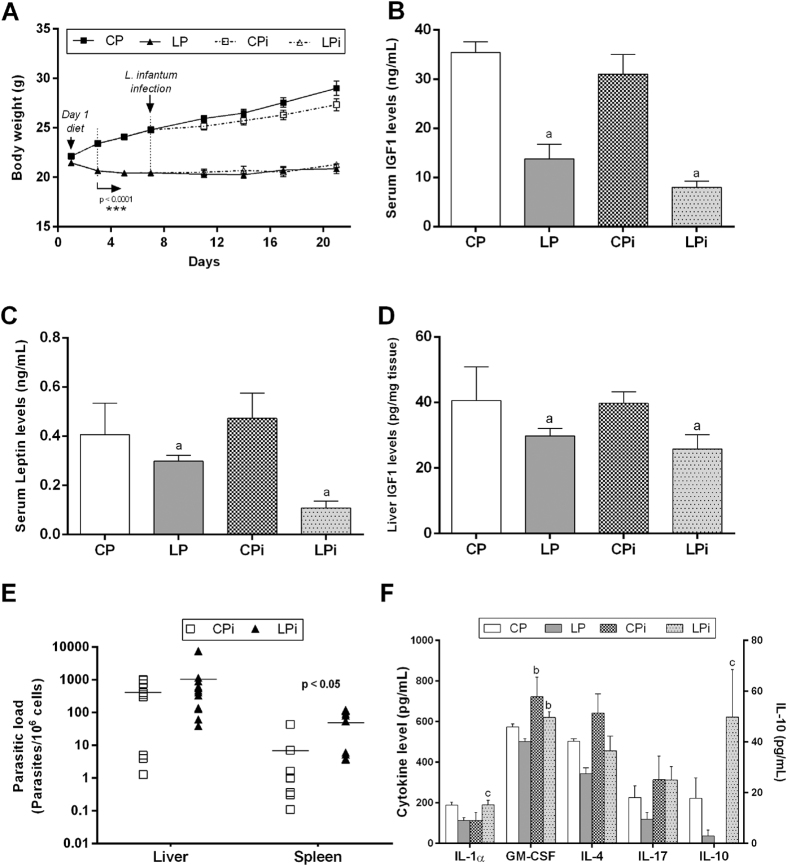
Body weight, hormonal levels, serum cytokine profile and parasite load in malnourished BALB/c mice infected with *L. infantum*. (**A**) Male BALB/c mice were fed a 14% (n = 24, CP) or 4% (n = 24, LP) protein diet for 21 days. On day 7 of the experimental period, half of the animals were infected with *L. infantum* and the other half received an injection of saline solution. Body weight was recorded every third day and expressed as average ± SEM; n = 12 mice in each group. Statistical differences before the day of infection were determined by Student’s *t* test (p < 0.0001). After infection, a Two-way ANOVA analysis with Bonferroni *post*-*hoc* test was used (p < 0.0001). Total IGF1 (**B**) and leptin (**C**) serum levels, as well as hepatic IGF1 (**D**) were measured by specific murine ELISA immunoassays. Parasite load was determined by qPCR in liver and spleen (**E**). The number of parasites was calculated in relation to 10^6^ cells. Data represent the mean of at least 10 animals in each group. Statistical differences were analyzed by Student’s *t* test (p < 0.01). (**F**) IL-1α, IL-4, IL-10, IL-17 and GM-CSF serum levels at 14 days post-infection were measured by flow cytometry using a Th1/Th2 multiplex assay. Statistical differences due to diet (a), infection (b) or an interaction between diet and infection (c) were determined by two-way ANOVA with Bonferroni *post*-*hoc* test (p < 0.05). CP: animals fed 14% protein diet; LP: animals fed 4% protein diet, CPi: animals fed 14% protein diet and infected; LPi: animals fed 4% protein diet and infected.

Male BALB/c mice were fed isocaloric control or low protein diets for 21 days. On day 7 of the experimental period, animals were randomly divided and half the animals were infected with *L. infantum*, setting four groups of study: **CP** (control protein): animals fed 14% protein diet; **LP** (low protein): animals fed 4% protein diet; **CPi** (control protein and infected): animals fed 14% protein diet and infected; **LPi** (low protein and infected): animals fed 4% protein diet and infected. Animals were euthanized 14 days post-infection (dpi). In agreement with previous reports^22^, food intake was comparable between CP and LP diet groups. Body weight differed significantly between the two dietary groups from day three of the diet (p < 0.0001) (Fig. 1A). At the day of infection (day seven of diet), and at day 21 (final day) LP mice had 17.5% (p < 0.0001) and 28.5% less body weight (p < 0.0001), respectively, compared to CP animals (Fig. 1A), indicating mild to moderate malnutrition^22^,^23^. As protein malnutrition is characterized by decreased levels of IGF1 and leptin we measured the levels of these molecules in our model. After a 21-day period of low protein intake, IGF1 serum levels in mice from the LP and LPi groups were significantly lower than those from the CP group (p < 0.0001, Fig. 1B). Similar to IGF1, leptin levels diminished in malnourished animals. Leptin levels from LP and LPi mice were significantly lower than those of CP animals after 21 days of the low protein diet (p < 0.05, Fig. 1C).

In order to determine if a low protein diet affects the main source of circulating IGF1, liver-derived IGF1 was also measured on the final day of the experiment (21 day of diet). Indeed, hepatic IGF1 levels were found significantly decreased in LP animals (p < 0.001) because of the protein restriction (Fig. 1D). In agreement with our previous report^22^, we observed an important reduction in the absolute thymocytes number in the thymus accompanied by a significant decrease of DP thymocytes concomitant with significant increase in CD4+ SP cells in the malnourished mice infected with *L. infantum* (Supplementary Fig. S1 and Supplementary Table S1).

### Malnourished mice exhibited higher splenic parasitic load than well-nourished animals

Liver and spleen samples were obtained from malnourished or well-nourished mice infected with *L. infantum*. Parasites were detected in the liver and the spleen by a qPCR assay with a detection limit of 0.1 parasites per 10^6^ cells, as described before^24^. qPCR determined that 100% liver and spleen samples were positive for the infection (Fig. 1E). Although not significant, the parasite load in the liver, ascertained by qPCR, was higher in LPi animals than in CPi ones (Fig. 1E). In addition, in agreement with our previous report, there was a significant increase in the parasite load in the spleen of LPi mice at 14 dpi (p < 0.05, Fig. 1E).

As an additional measure of the parasite visceralization, liver and spleen weight were assessed in each animal. Splenomegaly was observed in animals infected with *L. infantum*. (p < 0.05, Supplementary Fig. S2). In addition, the liver weight was significantly increased in protein restricted and infected animals (p < 0.05, Supplementary Fig. S2).

### Interleukin-1alpha and IL-10 serum levels are increased in protein malnourished BALB/c mice infected with *L. infantum*

In order to evaluate the cytokine serum profile of protein malnourished BALB/c mice infected with *L. infantum*, the levels of several cytokines were measured by flow cytometry using a Th1/Th2 multiplex assay. Malnourished mice exhibited a tendency for reduction of all analyzed cytokines due to protein malnutrition (Fig. 1F). However, a significant increase in IL-1α and IL-10 levels was observed in LP infected animals due to the interaction between protein malnutrition and infection (p < 0.05) (Fig. 1F). Interestingly, the levels of the granulocyte macrophage colony-stimulating factor (GM-CSF) were significantly higher in the *L. infantum* infected animals (p < 0.05) (Fig. 1F). Serum levels of IFN-gamma, TNF-alpha, IL-2, IL-5, and IL-6 were detected, but did not differ significantly among experimental groups (data not shown).

### Thymus is atrophied in infected malnourished animals but its weight is increased in well-nourished mice infected with *L. infantum* and parasites are detected in the thymus of infected animals

**Figure 2:**
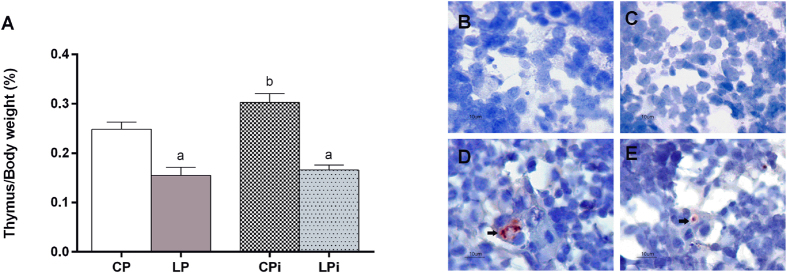
Effect of protein malnutrition on thymus weight in BALB/c mice infected with *L. infantum* and immunodetection of parasites. (**A**) Thymus weight at 14 dpi is expressed as a percentage of tissue/body weight in grams ± SEM (n = 10). Statistical differences due to diet (a), infection (b) were determined by two-way ANOVA with Bonferroni *post*-*hoc* test (p < 0.05). Amastigotes (arrows) were detected by immunohistochemistry (Red/AEC - 3-amino-9-ethylcarbazole). (**B**) CPi and (**C**) LPi thymus without anti-*Leishmania* antibody (Negative) and (**D**) CPi and (**E**) LPi thymus with anti-*Leishmania* antibody. Scale bar = 10 μm. CP: animals fed 14% protein diet; LP: animals fed 4% protein diet, CPi: animals fed 14% protein diet and infected; LPi: animals fed 4% protein diet and infected.

**Figure 3:**
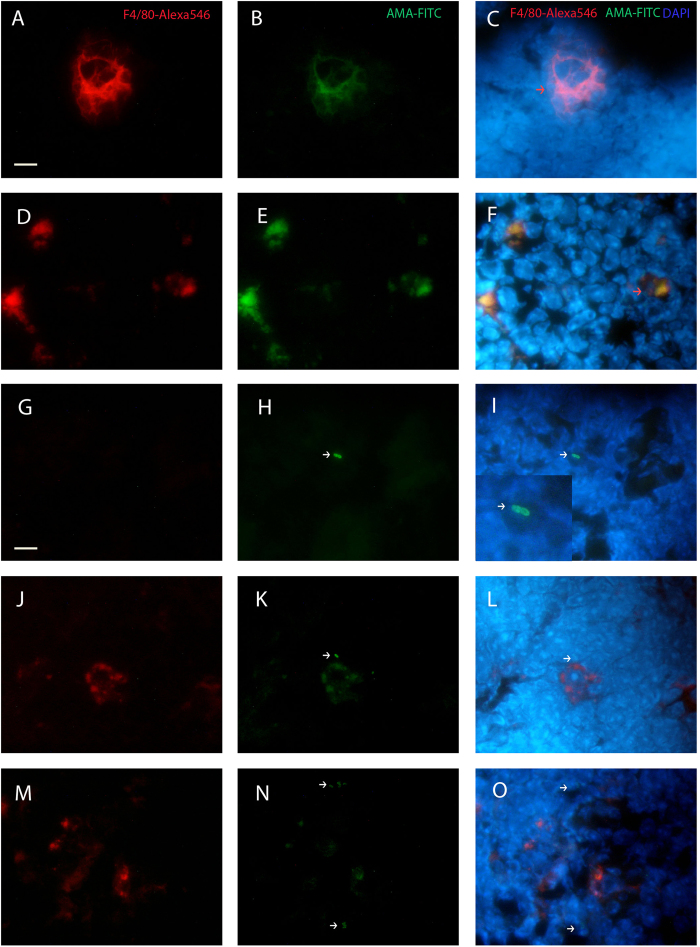
Colocalization analysis of F4/80^+^ macrophages and amastigotes in thymus by immunofluorescence. (**A–C**) CPi animal. (**D–O**) LPi animal. (**A**,**D**,**G**,**J**,**M**) Macrophages (red), (**B**,**E**) Amastigote antigens (green) and (**C**,**F**) macrophages with antigens in cytoplasm (orange arrow). (**G**,**H**,**I**) Two amastigotes inside F4/80^−^ cell (white arrow and zoom). (**J–L**) One amastigote (white arrow) close to a macrophage (F4/80^+^/red) containing *L. infantum* antigens in cytoplasm. (**M–O**) Two amastigotes nests outside macrophages (white arrows) and close to F4/80^+^ cells with and without *L. infantum* antigens in cytoplasm. (**C**,**F**,**I**,**L**,**O**) Merge. Bar = 10 μm.

Confirming our previous reports, the thymus of malnourished animals suffered a drastic weight loss relative to the body weight resulting in a significant loss of ~38% in the LP animals and ~34% in the LPi animals (p < 0.0001) compared to well-nourished animals at day 21 of diet (Fig. 2A). On the other hand, the weight of thymus of well-nourished animals increased 31% due to *L. infantum* infection (p < 0.05) (Fig. 2A). Such increase prompted us to evaluate whether *L. infantum* could be reaching the thymus in our model, thus we investigated the presence of the parasite in that tissue by immunohistochemistry and immunofluorescence. Surprisingly, parasites were detected in the thymus of both well-nourished and malnourished infected animals (Fig. 2B–E and Fig. 3). Intact amastigotes and/or amastigote antigens were observed more frequently in the medullary region, inside and outside the macrophages (F4/80+). Intact amastigotes were more commonly observed in LPi animals than the CPi and they appeared isolated or in groups forming amastigotes nests. Amastigote nests were only observed in LPi animals (Fig. 3O).

### Severe thymic atrophy in malnourished *L. infantum* infected mice is accompanied by modest increase in apoptosis of developing thymocytes

**Figure 4:**
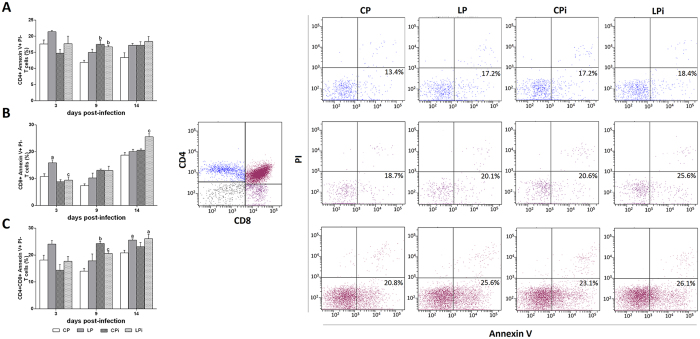
Apoptotic T cell subsets in the thymus of protein malnourished BALB/c mice infected with *L. infantum*. Annexin V positive CD4^+^ (**A**), CD8^+^ (**B**) or CD4^+^CD8^+^ (**C**) T cell subpopulations in the thymus were analyzed by flow cytometry. Apoptotic events of each lymphocyte subset are expressed as percentage ± SEM of cells binding Annexin V^+^-APC^−^ as described in the Methods. Left panels: Percentage of apoptotic cells by treatment. Right panels: Representative plots of T cell subpopulations expressing Annexin V at the 14 dpi (21 of diet). Upper panel: CD4^+^; middle panel: CD8^+^; lower panel: double positive T cells. Percentages in each panel indicate early apoptotic (lower right quadrant), late apoptotic (upper right quadrant), necrotic (upper left quadrant) cells. CP: animals fed 14% protein diet; LP: animals fed 4% protein diet, CPi: animals fed 14% protein diet and infected; LPi: animals fed 4% protein diet and infected. Two-way ANOVA with Bonferroni *post*-*hoc* test. Statistical differences due to diet (**a**, p < 0.05) and the interaction between diet and infection (**c**, p < 0.01).

As previously reported, protein malnutrition induced drastic thymic atrophy with a severe hypocellularity and particular decrease in CD4^+^CD8^+^ DP thymocytes of animals infected with *L. infantum*^22^. In the present study we corroborated that cellularity is drastically reduced in the thymus of malnourished and infected mice (Supplementary Table S1), thus, to determine if this reduction was related to an increase in cell death, we analyzed Annexin V and PI staining in thymocyte subsets. At 14 dpi, the percentage of apoptotic CD4^+^ single-positive (SP) T cells were no different between the groups (Fig. 4A), apoptotic CD8^+^ SP thymocytes increased by 7% in LPi animals due to the interaction of diet plus infection (Fig. 4B) and apoptotic DP cells increased modestly by ~5% in protein restricted animals at 14 dpi (Fig. 4C).

Potential apoptosis in thymocytes of protein malnourished BALB/c mice infected with *L. infantum* was also analyzed at the transcriptional level by evaluating mRNA expression levels of the pro-apoptotic genes *Caspase 3, Bax, Bid, Apaf1* (Supplementary Fig. S3). We also analyzed the expression of the anti-apoptotic genes *Bcl*-2 and *Survivin*. Quantitative data showed no significant differences in the expression of pro-apoptotic genes among groups (Supplementary Fig. S3). The expression levels of *Survivin* decreased significantly due to protein malnutrition (p < 0.01), whereas the anti-apoptotic gene *Bcl*-2 exhibited a significant increase in expression levels in the thymuses from protein malnourished animals (p < 0.01, Supplementary Fig. S3).

### *Leishmania infantum* infection modulates intrathymic expression of CXCR3 and ligands

**Figure 5:**
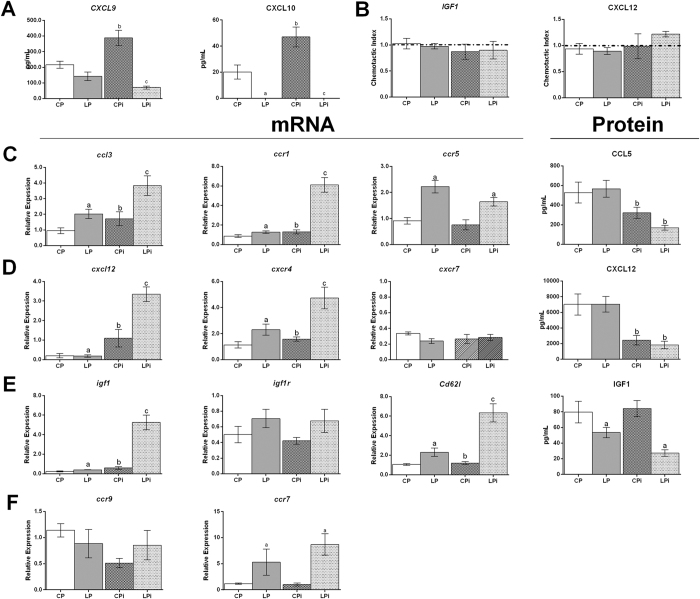
*Ex*-*vivo* directed migration in response to IGF1 and CXCL12 of thymocytes from protein malnourished BALB/c mice infected with *L. infantum* and mRNA or protein levels of chemotactic molecules involved in cell migration. (**A**) Secreted CXCL9 and CXCL10 were quantified by Luminex in the interstitial fluid of the thymus of each experimental group. The values are expressed in pg/mL ± SEM. (**B**) Thymocytes were stimulated with IGF1 or CXCL12 and cell migration was expressed as a chemotactic index related to the cells that migrated in the absence of stimuli. (**C**,**D**,**E**,**F**) *Ccl3, Ccr1, Ccr5, Cxcl12, Cxcr4, Cxcr7, Igf1, Igf1r, Cd62l, Ccr7* and *Ccr9* mRNA expression levels and CCL5, CXCL12 and IGF1 protein levels. mRNA levels were measured by qPCR in thymocytes of each experimental group. The values are expressed as normalized ratios between the target gene expression and the geometric median of the genes *Atp*-5, *Gapdh* and *Cyc*-1. CCL5, CXCL12 and IGF1 protein levels were determined by ELISA in the interstitial fluid of the thymus and are expressed as pg/mL. CP: animals fed 14% protein diet; LP: animals fed 4% protein diet, CPi: animals fed 14% protein diet and infected; LPi: animals fed 4% protein diet and infected. Two-way ANOVA with Bonferroni *post*-*hoc* test. Statistical differences due to diet (**a**, p < 0.05), infection (**b**, p < 0.05) and the interaction between diet and infection (**c**, p < 0.05).

In order to determine if defects in cell migration could account for the drastic decrease in thymus cellularity, we quantified the expression of the chemokine receptor CXCR3 on thymocyte subsets as well as the levels of its ligands CXCL9 and CXCL10. At 14 dpi, the expression of CXCR3 showed a significant increase in the CD8^+^ SP T cells from infected animals (p < 0.05) (Supplementary Fig. S4). Remarkably, the secreted protein levels of CXCL9 and CXCL10 increased in well-nourished infected animals (p < 0.01), but decreased significantly in malnourished infected mice (p < 0.01, Fig. 5A).

### *Ex*-*vivo* thymocyte migratory capabilities are preserved but protein levels of chemotatic ligands are significantly reduced in the thymus of malnourished and infected animals

The significant reduction of CXCL9 and CXCL10 protein levels observed here (Fig. 5A) prompted us to evaluate the stimuli-driven migratory capabilities of thymocytes in our model. In order to verify such capability, we carried out an *ex*-*vivo* cell chemotaxis assay exposing thymocytes to CXCL12 or IGF1 stimulus. Besides CXCL12, we analyzed IGF1 because it has been previously demonstrated its role on regulating cell migration^25^ and, particularly, a protecting effect under nutritional stress, through the maintenance of cell proliferation and migration^26^. Remarkably, thymocytes from the different animal groups did not exhibit changes in the chemotactic migration index, neither when exposed to CXCL12 nor IGF1 (Fig. 5B), reinforcing the observation that malnutrition has a deleterious effect on the thymic microenvironment, mostly on the production and secretion of chemokines, instead of affecting T cell migratory capabilities *per se*.

Thus, to further address the hypothesis that protein malnutrition could be dysregulating the expression of chemotactic factors, we analyzed, by qPCR, mRNA gene expression of chemotactic related genes as a measurement of potential alterations in migration capability. The mRNA expression levels of the chemokines *Ccl3* and *Cxcl12* and the levels of *Igf1* in thymocytes were clearly upregulated due to protein malnutrition or infection conditions separately (Fig. 5C–E) (*Ccl3* p < 0.01; *Cxcl12* p < 0.05 and *Igf1* p < 0.001). Remarkably, due to an additive or synergic effect, the interaction between both variables (malnutrition and infection) significantly increased the mRNA levels of these thymic chemokines (p < 0.05) (Fig. 5C–E). Accordingly, the expression of the receptors for *Ccl3* and *Cxcl12, Ccr1* and *Cxcr4*, respectively, were significantly upregulated by the interaction of low diet and infection (p < 0.05) (Fig. 5C and D). *Ccr5*, another receptor for *Ccl3*, was only upregulated due to malnutrition (p < 0.0001) (Fig. 5C). Furthermore, the expression levels of the receptor for IGF1 (*Igf1r*) increased by both the protein diet restriction alone and combined with infection, although the differences were not significant (Fig. 5E). Since cell migration depends on the interaction between adhesion molecules, we decided to explore if malnutrition could be affecting the expression of *Cd62l (L*-*selectin*). Indeed, protein malnutrition significantly increased the expression of this gene in thymocytes of infected animals (p < 0.01) (Fig. 5E).

In addition, the protein levels of CCL5 and CCL3, as ligands of CCR5 and CCR1, as well as the protein levels of CXCL12, ligand of CXCR4 and CXCR7, were measured in the interstitial fluid of the thymus (Fig. 5C and D, Protein column). Protein levels of CCL3 were not detected in the interstitial fluid of the thymus even when using a very sensitive methodology such a Luminex (data not shown). Noticeably, the concentration of CCL5 and CXCL12 decreased significantly due to the infection in the thymus (Fig. 5C and D, CCL5 p < 0.001; CXCL12 p < 0.0001). Remarkably, IGF1 reduced significantly in thymic interstitial fluids due to protein malnutrition (Fig. 5E, p < 0.001).

Finally, the expression of *Ccr7*, a chemokine receptor involved in SP thymocyte migration towards the medulla, was significantly upregulated due to malnutrition (p < 0.001). Remarkably, such increased expression was twice higher in malnourished animals infected with *L. infantum* than in malnourished-non-infected mice (p<0.001). *Ccr9* expression levels did not show significant changes among the groups (Fig. 5F).

## Discussion

We previously demonstrated that the interaction between protein malnutrition and *L. infantum* infection resulted in significant thymic atrophy, reduced cellularity and changes in thymic T cell subsets in BALB/c mice and led to increased splenic parasite load, which was accompanied by a drastic loss of spleen microarchitecture^22^. A characteristic of this model of moderate malnutrition was the significant alteration of thymic T cell populations and reduction of DP T cells due to the synergy of protein malnutrition and *L. infantum* infection, and an intriguing role of the parasite *per*-*se* in those alterations^22^. Herein, the loss of thymic mass as a result of reduced cellularity was accompanied by significant alterations in the expression, at mRNA and protein levels, of chemokynes, IGF1 and adhesion molecules involved in T cell migration in the thymus and increased parasite load in the spleen. Furthermore, molecules involved in apoptosis were not significantly altered in this model. These data provide new information about the effect of malnutrition in the thymic microenviroment during infection with *L. infantum* and show that parasites were detected in the thymus of infected animals.

Thymic atrophy with reduced cellularity under malnutrition or infection conditions has been attributed to increased T cell apoptosis and reduced T cell proliferation^27^,^28^,^29^. In our model, the percentage of apoptosis in the DP T cells just increased 5% at 14 dpi due to the protein restricted diet. Additionally, pro-apoptotic gene expression remained unaltered among the experimental groups. These results contrast with previous reports on the effect of malnutrition on DP cells that showed that apoptosis is responsible for the DP T cell depletion in the thymus^15^. Nevertheless, we cannot discard that apoptosis could have occurred in earlier stages of malnutrition and/or infection in our model, and at day 21 of diet other mechanisms have arisen to counteract the deleterious effect of malnutrition. Interestingly, the anti-apoptotic gene *Bcl*-2 was upregulated by the protein restricted diet at 14 dpi. This is in accordance with other malnourishment models where BCL-2 protein was overexpressed in thymocytes of protein deprived mice, thereby protecting them from apoptotic stimuli^30^. In fact, *Bcl*-2 is a molecule induced by CXCR4 signalling^31^ and we observed here that increased expression of *Bcl*-2 correlates with increased expression of *Cxcr4*. A significant reduction in *Survivin* gene expression in the thymocytes of protein-restricted animals was observed. Survivin is an inhibitor of apoptosis and also has an important role in mitosis^32^. In the thymus, the role of survivin is mainly associated with cell proliferation at specific checkpoints during T cell maturation^33^. The lack of survivin expression resulted in a reduced number of peripheral T cells and egress of immature T cells, but it did not leed to an increase in apoptosis^33^,^34^. Decrease in intrathymic cell proliferation during malnutrition has been previously reported^17^. Our results show that the protein restricted diet induced reduced cellularity^22^ and reduced *Survivin* gene expression in the thymus, suggesting that reduced expression of this protein would impact the T cell development and homeostatic expansion; however, the molecular bases of this event remain unexplored. Together, our results suggest that variables other than apoptosis, such as intrathymic proliferation, migration or cell entry into the thymus, may be influencing the thymic atrophy. On-going experiments will determine this issue.

To explore if changes in cell migration capability could contribute to thymic cell depletion in this model, we first determined CXCR3 expression on T cell subsets in the thymus of mice and the levels of the ligands CXCL9 and CXCL10 in the interstitial fluid (IF) of the organ. In the human thymus, at least 4 subset of thymocytes express CXCR3: TCRab^+^CD8^+^ single positive T cells, TCRγδ^+^ T cells, a small subset of CD1a^+^CD4^+^CD8^+^ T cells, and cells showing the natural killer (NK)–type phenotype^35^. In the thymus of mice we found that CXCR3 is expressed in all three of the evaluated thymic subsets: CD4^+^ and CD8^+^ SP T cells and CD4^+^CD8^+^ DP T cells. Although *L. infantum* infection modulates modestly the percentage of CXCR3^+^CD8^+^ T cells, it remarkably increases the levels of CXCL9 and CXCL10 in the thymus. This result represents the first report, to our knowledge, of the influence of *L. infantum* infection on the expression of chemokines in the thymus. Increased levels of CXCL9 and CXCL10 were observed in the thymuses of mice infected with *Mycobacterium tuberculosis*^36^. The increase in these chemokynes induced the recruitment of CXCR3^+^ peripheral T cells back to the thymus for intrathymic bacterial control. In our model, well-nourished mice infected with *L. infantum* presented also a significant increase in protein levels of CXCL9 and CXCL10, probably promoting recirculation of mature SP CXCR3^+^ peripheral T cells back to the thymus to respond to thymic infection. In contrast, malnourished animals infected with *L. infantum* were not able to increase the levels of these chemokynes, suggesting a further defect in cell migration, in this case, back to the thymus, for controlling infection.

On other hand, the ligands CXCL9 and CXCL10 play an important role in the transmigration of mature thymocytes during the process of lymphopoiesis in the thymus^35^. Production of these chemokines in distinct thymic areas indicated that they may selectively act on distinct thymocyte subsets according to their different receptor affinity^35^. Therefore, in a microenvironment with reduced levels of these chemokines, as the one observed here in malnourished animals, the intrathymic migration of CXCR3^+^ T cells should be affected. Thus, we hypothesized that during protein malnutrition and *L. infantum* infection, the impairment of the CXCL9/CXCL10-CXCR3 axis could negatively impacts (i) the recirculation of mature SP CXCR3^+^ peripheral T cells back to the thymus to respond to *Leishmania* thymic infection or (ii) the thymocyte maturation and the migration of different subsets of mature thymocytes.

Additionally, we analyzed mRNA expression levels of some chemotactic genes. We observed that protein malnutrition induced a significant increase of mRNA expression of the genes *Ccl3, Ccr1, Ccr5, Cxcl12, Cxcr4*, and *Igf1* in thymocytes of *L. infantum* infected mice. CCL3 protein levels were undetectable in the interstitial fluid of the thymus, however, CCL5, another ligand of CCR5, was detected in thymic IF in all experimental groups, although their levels were significantly reduced in infected animals. In agreement, during thymic atrophy associated with age or infection, an increase in mRNA expression of CCR5 has been reported^18^,^37^. CCR1 has not been explored in the thymus, but the expression of CCR5 and CCL5 have been documented in this organ^37^,^38^. As CCR5 controls thymic egress of Tregs during infection^39^ and it has been showed that during cutaneous leishmaniasis CCR5-dependent homing of Tregs regulates the proinflammatory response and favor pathogen persistence^40^, it is plausible to suggest that increase in CCR5 expression could induce increased migration (egress from thymus) to inflammatory sites, favoring parasite persistence in secondary lymphoid organs such as spleen. However, we are aware that we are working with a heterogeneous pool of thymocytes that may include, among the SP population, NKT, Treg, γδ, and recirculating memory αβ T cells. In young mice, as the ones used in our model, such cells typically comprise 10–15% of the SP pool^41^, but we cannot state exactly which proportion of SP cells were nTregs in malnourished animals. The functions of CCL5 in the thymus have not been well described, but they are known to be associated with enhanced migration and adhesion of different cell types^37^,^42^. It is possible that the reduction of CCL5 in our model impacts intrathymic migration or entry into the thymus during *L. infantum* infection in order to minimize parasite persistence in this organ.

It has been shown that *Igf1* and its receptor are expressed by human and murine thymic epithelial cells (TEC) and thymocytes and that *Igf1* mediates the pleiotropic effects of growth hormone in the thymus including production of extracellular matrix components and increasing cell adhesion^17^. Interestingly, in our study the increase of *Igf1* mRNA levels in malnourished and infected mice was not followed by an increase in protein levels locally in the thymus or systemically in serum, indicating a defect in protein synthesis by this growth factor. Such a defect could have a deleterious impact on the production of the extracellular matrix by TEC as well as on thymocyte adhesion and differentiation. However, in our model, thymocyte migratory capabilities were preserved, suggesting that protein malnutrition compromises the thymic microenvironment (instead of T cell migration capability *per se*), affecting soluble factors in the extracellular matrix, cell adhesion and ultimately cell migration through the thymic epithelium. These results are in keeping with previous demonstrations that the thymic microenvironment is a main target in malnutrition^19^,^43^,^44^ as well as during infection with another trypanosomatid, namely *Trypanosoma cruzi*^18^,^45^,^46^, as well as other infectious agents^16^,^47^.

For proper T cell differentiation, the CXCL12-CXCR4 axis mediates the flow of primarily immature thymocytes from the sub-capsular to the medullar region of the thymus^48^,^49^. In *T. cruzi* and *P. berguei* infection models, increased abundance of CXCL12 and CXCR4 is associated with an enhanced migratory response of thymocytes^18^,^50^. We observed an increase in mRNA expression of these molecules, but a significant reduction of CXCL12 protein levels in the IF of the thymus during protein malnutrition and *L. infantum* infection. Nevertheless, *ex*-*vivo* assays showed that neither *L. infantum* infection nor diet altered the migratory responses of thymocytes triggered by CXCL12 or IGF1. Since the interaction of CXCL12 with components of the extracellular matrix in the thymus seems crucial for thymocyte migration^18^,^51^, it is plausible to suggest that a low abundance of CXCL12, plus thymic disorganization due to malnutrition, can affect that interaction. The same scenario could be true for IGF1 and its interaction with integrins, as has been observed with other tissues^52^. In fact, the increase of *Cd62l* (formerly *l*-*selectin*) expression in malnourished and infected animals, concomitantly with accumulation of CD4^+^SP cells, could also indicates an alteration in cell adhesion in the thymus. Thus, changes in the expression of *Igf1*, chemokines and adhesion factors, together with thymic atrophy, strongly suggest that the interaction of thymocytes with the components of the extracellular matrix and the thymic microenvironment are altered in protein malnourished and infected animals. However, further structural studies should be conducted to understand the role of protein malnutrition in extracellular matrix organization, adhesion molecules and T cell maturation during *L. infantum* infection.

It has been also showed that positively selected CD8^+^ or CD4^+^ SP cells upregulate *Ccr7* and migrate to the medulla in response to CCL19/CCL21 (produced by mTECs, and endothelial venules in the medulla)^53^. After complete their maturation SP cells leave the thymus and enter the peripheral circulation^41^. Knockout of *Ccr7* was associated with accumulation of SP thymocytes in cortex and decrease of CD4^+^SP and CD^+^8 SP in medulla^54^,^55^. Also, it was demonstrated that treatment with an inhibitor of thymocyte migration induced accumulation of *Ccr7*-wild type SP cells in medulla^55^. In our study, increased levels of *Ccr7* in malnourished animals occurred along with increased levels of *Cd62L*, another phenotypic marker of mature SP cells, suggesting an accumulation of SP thymocytes in the thymus in malnourished animals. Such results agree with the significant increasing of CD4^+^ thymocytes subpopulation in the thymus of malnourished animals^22^, this work. Thus, one potential explanation for the significant DP depletion in our malnourished model would involve rapid maturation and accumulation of thymic CD4^+^ SP cells, demonstrated by the significant increase in *Ccr7* and *Cd62L* and significant increase in CD4^+^ thymocytes.

Another hypothesis for explaining DP depletion would involve the arrest of DN to DP transition. In this context, it was demonstrated that *Ccr7* is expressed by transitional DN1-2 thymocytes and knockout of *Ccr7* gene in mice induced accumulation of DN2 thymocytes at the cortico-medullary junction^56^. In addition, *Ccr7*-deficient mice exhibit decreased absolute numbers of thymocytes and altered thymic architecture^56^. As DN1 and DN2 thymocytes also express *Ccr7*, we cannot rule out the possibility that in malnourished animals part of the DN thymocytes did not complete their transition to DP cells staying at an immature form of DN1-2 cells. Such a hypothesis would explain another proportion of decreased DP cells observed in malnourished animals. However, such hypothesis remains to be explored.

In summary, the thymic microenvironment revealed reductions in important chemotactic molecules such as CCL5, CXCL12, IGF1, CXCL9 and CXCL10 during protein malnutrition and *L. infantum* infection in BALB/c mice. The reduced levels of these molecules suggest that (i) the progenitor’s entry into the thymus and migration could be compromised; (ii) the control of the infection in the thymus, mediated by recirculation of mature SP CXCR3^+^ peripheral T cells back to the thymus could be also compromised; (iii) the increase of *Ccr7* and *Cd62l* levels on thymocytes from malnourished animals concomitant with the accumulation SP cells observed on these animals, reinforce the hypothesis that protein malnutrition has a deleterious defect on thymocyte migration. Together, these events result in thymic atrophy and premature disease at secondary lymphoid organs, such as the spleen, by affecting proper T cell responses involved in the control of *L. infantum* infection. In fact, herein as well as in our previous work we observed that BALB/c mice subjected to a low protein diet and *L. infantum* infection suffered a drastic disruption of splenic architecture associated with increased parasite load^22^.

Together, our findings show that protein restriction leads to a local reduction of chemotactic factors in the thymus of BALB/c mice infected with *L. infantum* followed by an increased parasite load in the spleen. Our data showed that during protein malnutrition and *L. infantum* infection, the thymus suffered drastic dysregulation of migratory molecules that can impact intrathymic and peripheral T cell motility and suggest that the entry/exit of different types of cells including immature and mature lymphocytes is affected. Our findings highlight the role of the local microenvironment of thymus in the control of *L. infantum* infection when a malnutrition condition pre-exists, and suggest that such a precondition has a deleterious effect on T cell-mediated immune responses by interfering with cell migration and thus reducing the capacity of protein-deprived animals to control parasite proliferation.

## Methods

### Ethics statement

This study was carried out carried out in accordance with the recommendations in the Guide for the Care and Use of Laboratory Animals of the National Institutes of Health - Eighth Edition. All animal procedures were approved by the Instituto Oswaldo Cruz Animal Care and Use Committee (License # LW-27/14). The *L. infantum* strain MCAN/BR/2000/CNV-FEROZ used in this study was provided by the Collection of *Leishmania* of the Instituto Oswaldo Cruz, (Coleção de *Leishmania* do Instituto Oswaldo Cruz, CLIOC) (http://clioc.fiocruz.br/). CLIOC is registered in the World Federation for Culture Collections (WFCC-WDCM 731) and is recognized as a Depository Authority by the Brazilian Ministry of the Environment (D.O.U. 05.04.2005).

### Parasite culture

Parasites were cultured at 25 °C in Schneider’s medium containing 10% fetal bovine serum (FBS) and were collected at the stationary phase by centrifugation at 1800 g for 5 min. The parasites were then washed twice in PBS, pH 7.2.

### Mice, feeding protocol and experimental infection

BALB/c mice (n = 48) were weaned at postnatal day 21 and given a diet containing 14%-protein (MP Biomedicals, Inc., USA, Catalog No 960258). Mice were maintained in 12/12 h light/dark cycle. After 1-week acclimation, animals were randomly divided into two groups with 24 animals being fed 14% (control protein, CP) and 24 animals being fed 4% protein content diet (low protein, LP) (MP Biomedicals, Catalog No 960254). These two diets are isocaloric with each providing 3.7 kcal/g. The animals had free access to water and food. After 7 days of diet, each animal group was divided into two subgroups, and one subgroup of each diet group was infected intravenously (tail vein) with 1 × 10^7^ parasites, whereas the other two subgroups received saline. The resultant four study groups were: animals fed 14% protein diet (CP), animals fed 4% protein diet (LP), animals fed 14% protein diet and infected (CPi), and animals fed 4% protein diet and infected (LPi). Diets were maintained after infection. Body weight was recorded every third or fourth day, but mice were monitored daily during the course of infection (14 days). Animals were sacrificed at 14 days post infection (dpi). Blood was collected by cardiac puncture and sera were separated and stored at −30 °C. The spleen, thymus, and liver were quickly removed, weighed, and subsequently processed for cell isolation or nucleic acid extraction.

### Parasite load

Positivity of infection and parasite load in the liver and spleen were measured by real-time quantitative PCR (qPCR) following procedures previously described^22^,^24^. In brief, total DNA was extracted from the liver and spleen using the Wizard Genomic DNA Purification System (Promega, Madison, WI, USA), which includes a prior digestion phase with 17.5 μl of proteinase K (20 mg/mL) for 12 h at 55 °C. DNA was dissolved in 100 μl of Tris-EDTA buffer. Parasite load was estimated by qPCR in samples using a kDNA target and the ubiquitin C (UBC) gene for normalizing murine DNA concentrations in each sample, followed by an adjustment to cell number.

### Hormone and chemokine levels

Using specific enzyme-linked immunosorbent assays, systemic and local total IGF1 was measured in serum and liver, respectively; IGF1 was also measured in interstitial fluid of the thymus (obtained by gentle intra-thymic washing) and leptin levels were measured in serum (Abcam Cambridge, UK; Cat.# ab100695 and ab100718, respectively) according to the procedures provided by the manufacturer. Levels were expressed in ng/mL for serum, pg/mL for fluid and pg/mg tissue for the liver. Chemokine levels of soluble ligands such as CCL5 and CXCL12 were measured in interstitial fluid of the thymus using a DuoSet^®^ELISA development system (R&D Systems, Cat. DY478 and DY460, respectively) according to the manufacturer’s protocols. Levels were expressed in pg/mL. Other soluble chemokines such as CCL3, CXCL9 and CXCL10 were quantified in the thymic interstitial fluid using Luminex technology^57^,^58^. The fluorescence levels of each molecule were measured and the data were analyzed using the software supplied by the manufacturer (Invitrogen). A series of recombinant cytokines applied in concentrations ranging from 51 to 8,000 pg/mL were used to establish standard curves and assay sensitivity. Levels were expressed in pg/mL.

### Serum cytokine levels

Serum samples were analyzed for the presence of cytokines (IL-1alpha, IL-2, IL-5, IL-6, IL-10, IFN-gamma, TNF-alpha, granulocyte monocyte colony-stimulating factor (GM-CSF), IL-4 and IL-17) using a FlowCytomix mouse Th1/Th2 kit (Bender MedSystems) according to the manufacturer’s instructions. Briefly, fluorescent beads coated with antibodies against to the specified cytokines were added to serum samples and incubated with biotinylated secondary antibodies. The amount of cytokine that bound to the antibodies was recognized by streptavidin-phycoerythrin conjugate and then detected using a flow cytometer (FACScan, Becton Dickinson, San Diego, CA, USA). Cytokine levels were determined by comparison with a standard curve (concentration range for all cytokines 0-20000 pg/mL) using FlowCytomix Pro 2.4 software.

### Immunohistochemistry and immunofluorescence

Thymus fragments frozen in OCT resin (Sakura) were cut into 3–5 μm thick sections and mounted on microscope silanized slides (Dako Cytomation, CA, USA). Slides were fixed in acetone PA (Merck, Darmstadt, Germany) and sequentially treated with peroxidase blocking reagent (Dako) and 0.4% BSA for blocking endogenous peroxidase and nonspecific staining, respectively. The slides were incubated with an anti-*L. infantum* rabbit antibody (1:200, kindly donated by Dr. Rodrigo C. Menezes), followed by incubation with an anti-rabbit IgG-HRP-conjugated antibody. Aminoethylcarbazole (AEC, Invitrogen) was used as the substrate–chromogen developing system and the slides were counterstained with Mayer’s hematoxylin (Sigma).

Thymus fragments were obtained by cryosection and prepared for immunofluorescence microscopy for amastigotes and macrophages detection and colocalization. Briefly, slides were rehydrated with PBS (pH 7.4). Unspecific epitopes were blocked with 0.4% BSA and then the sections were incubated with rat anti-mouse F4/80 (Abcam) and anti-*L. infantum* polyclonal rabbit serum, as primary antibodies. Goat anti-rabbit FITC-conjugated (eBioscience), Goat anti-Rat Alexa Fluor-546 (Calbiochem) were used as secondary antibodies. After staining, sections were mounted in medium containing DAPI (Fluoromount-G, eBioscience). Immunofluorescence was analysed in a Nikon Eclipse E400 Microscopy (Nikon digital camera DXM1200F and Nikon ACT-1 software, Nikon, Japan). The images were processed using the program ImageJ (NIH, USA).

### Flow cytometry analysis

For analysis of lymphocyte subpopulations, thymocytes were obtained by PBS washes of the respective organs. One million cells (1 × 10^6^) were immunostained with anti-mouse CD3 PE-conjugated, CD4 FITC-conjugated, or CD8a PECy7-conjugated antibodies (Becton Dickinson) or with IgG isotype-matched control antibodies. Acquisition (10,000 events) was performed in a FACSCanto™ flow cytometer. Off-line analysis was performed with BD FACSDIVA^TM^ Software, version 4.0. For analyzing the percentage of apoptotic cells, CD4^+^, CD8^+^ and DP T cells were stained with Annexin V-APC conjugated by the addition of 5 μl of Annexin V (BD Biosciences) at room temperature. After incubation in the dark at room temperature for 10 min, cells were incubated with PI. Annexin V/PI stained cells were then analyzed by flow cytometry as previously described. In addition, we measured the relative numbers of T cells expressing the chemokine receptor CXCR3 by flow cytometry (using an APC-conjugated anti-CXCR3 monoclonal antibody).

### Gene expression analysis

Total RNA was extracted from cells using Trizol Reagent (Life Technologies, Inc) according to the manufacturer’s instructions. RNA was quantified with a Nanodrop ND-1000 spectrophotometer (NanoDrop Technologies). cDNA was synthesized from 1 μg of total RNA using SuperScript III reverse transcription system (Invitrogen). Real time PCR was performed in duplicate using StepOne equipment and SYBR^®^ Green PCR Master Mix (Applied Biosystems), according to the manufacturer’s protocol. Oligonucleotide sequences of the target genes *Bcl*-*2, Bax, Bid, Caspase 3, Apaf1, Survivin*, C*cl3, Cxcl12, Ccr1, Cxcr4, Ccr5, Cxcr7, Ccr7, Ccr9, Igf1, Igf1r* and *Cd62l* in addition to reference genes *Gapdh, Atp*-*5, Cyc*-*1*, and *Ubc* (Supplementary Table S2) were designed from available public sequences using Primer Blast software. PCR conditions for all primers were optimized and specificities were verified by melting curve analyses and agarose gel electrophoresis. cDNAs were amplified by PCR for 40 cycles consisting of 10 s of denaturation at 95 °C, 15 s of annealing at 56 °C (see Supplementary Table S2), and 10 s of extension at 72 °C. Standard curves for all genes were generated using serial dilutions of pooled cDNAs from all samples. The efficiency of amplification for each gene was determined using the manufacturer’s software during the exponential phase of amplification. Gene expression was quantified by means of the comparative Ct method (ΔΔCt). Data are shown as normalized ratios between target gene expression and geometric media of the three reference genes^59^. Experiments were performed following the MIQE guidelines^60^.

### Chemotaxis assays

Thymic lymphocyte suspensions were prepared as described above. Approximately 10^6^ cells were resuspended in 0.2 mL of PBS supplemented with 1% BSA and placed in the upper chamber of Transwell inserts (5 μm pore size; Corning Costar, Corning). Inserts were placed in wells containing medium alone (basal) or medium plus: 100 ng/mL CXCL12 (R&D Systems Inc., MN, USA), or 10 nM IGF1 (GroPep Inc., Australia). Stimuli were applied at optimal concentrations determined by previous titration. After 3 h of migration at 37 °C, inserts were removed, and cells in the lower chamber were harvested and stained with anti-mouse CD3 APC-conjugated, CD4 FITC-conjugated, or CD8a PerCP-conjugated antibodies (Ebiosciences) or by the isotype-matched IgG control antibodies. Acquisition (10,000 events) was performed in a FACScan flow cytometer. Off-line analysis was performed with Summit Software, version 4.0. Cell migration was expressed as a chemotactic index related to the number of cells migrating in the absence of stimuli.

### Statistical analysis

Statistical analysis was performed using GraphPad Prism 6.0 software. A Two-way analysis of variance (ANOVA) was used, in conjunction with Bonferroni *post*-*hoc* test, to analyze differences among treatments. According to this analysis, significant differences (p > 0.05) due to diet, infection or an interaction between diet and infection are denoted with *a, b*, or *c* at each figure. The Student *t* test was used to analyze differences in body weight due to diet treatments (CP and LP) before infection, and to analyze differences in parasite load of CPi and LPi animals. The data are presented as the mean ± SEM.

## Additional Information

**How to cite this article**: Losada-Barragán, M. *et al*. Protein malnutrition promotes dysregulation of molecules involved in T cell migration in the thymus of mice infected with *Leishmania infantum. Sci. Rep.* **7**, 45991; doi: 10.1038/srep45991 (2017).

**Publisher's note:** Springer Nature remains neutral with regard to jurisdictional claims in published maps and institutional affiliations.

## Supplementary Material

Supplementary Information
