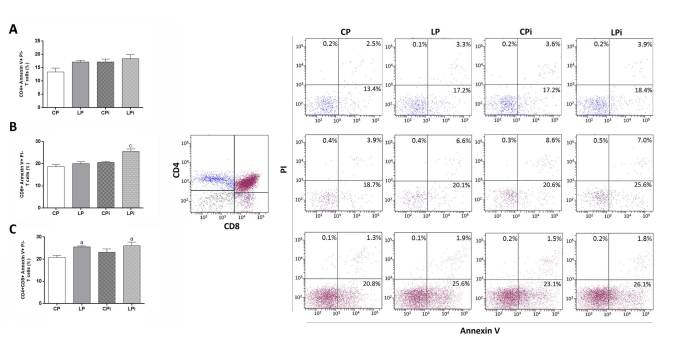# Erratum: Protein malnutrition promotes dysregulation of molecules involved in T cell migration in the thymus of mice infected with *Leishmania infantum*

**DOI:** 10.1038/srep46809

**Published:** 2017-09-25

**Authors:** Monica Losada-Barragán, Adriana Umaña-Pérez, Sergio Cuervo-Escobar, Luiz Ricardo Berbert, Renato Porrozzi, Fernanda N. Morgado, Daniella Areas Mendes-da-Cruz, Wilson Savino, Myriam Sánchez-Gómez, Patricia Cuervo

Scientific Reports 7: Article number: 45991; 10.1038/srep45991 published online: 04 11 2017; updated: 09 25 2017.

In this Article, Figure 4 is incorrect. The correct Figure 4 appears below as Figure 1. The legend of Figure 4 was correct from the time of publication.

## Figures and Tables

**Figure 1:**