# Supplementary material for: Protein malnutrition promotes dysregulation of molecules involved in T cell migration in the thymus of mice infected with Leishmania infantum
Source: Sci Rep. 2017 Apr 11;7:45991. doi: 10.1038/srep45991 (PMC5387407; doi:10.1038/srep45991)
Supplement: Supplementary Information [file srep45991-s1.pdf]

Protein malnutrition promotes dysregulation of molecules involved in T cell migration in the thymus of mice infected with *Leishmania infantum*

Monica Losada-Barragán[1¶], Adriana Umaña-Pérez[2¶], Sergio Cuervo-Escobar[2], Luiz Ricardo Berbert[3], Renato Porrozzi[1], Fernanda N. Morgado[1]; Daniella Areas Mendes-da-Cruz[3], Wilson Savino[3], Myriam Sánchez-Gómez[2]*, Patricia Cuervo[1]*

[1]Laboratório de Pesquisas em Leishmaniose, Instituto Oswaldo Cruz, Fiocruz, Rio de Janeiro, RJ, Brasil;

[2]Universidad Nacional de Colombia, Sede Bogotá, Facultad de Ciencias, Departamento de Química, Grupo de Investigación en Hormonas, Bogotá, Colombia;

[3]Laboratório de Pesquisas sobre o Timo, Instituto Oswaldo Cruz, Fiocruz, Rio de Janeiro, RJ, Brasil

Supplementary Figure S1

Effect of protein malnutrition of lymphocyte subpopulations in the thymus of mice infected with *L. infantum*. (**A, B, C**) Representative scatter plots of lymphocyte subpopulations. (**D**) Distribution of lymphocyte subsets expressed as percentage ± SEM. CP: animals fed 14% protein diet; LP: animals fed 4% protein diet, CPi: animals fed 14% protein diet and infected; LPi: animals fed 4% protein diet and infected. Two-way ANOVA analysis with Bonferroni pos-hoc test. Statistical differences due to diet: a ($p<0.05$), infection: b ($p<0.05$) and interaction between diet and infection: c ($p<0.05$).

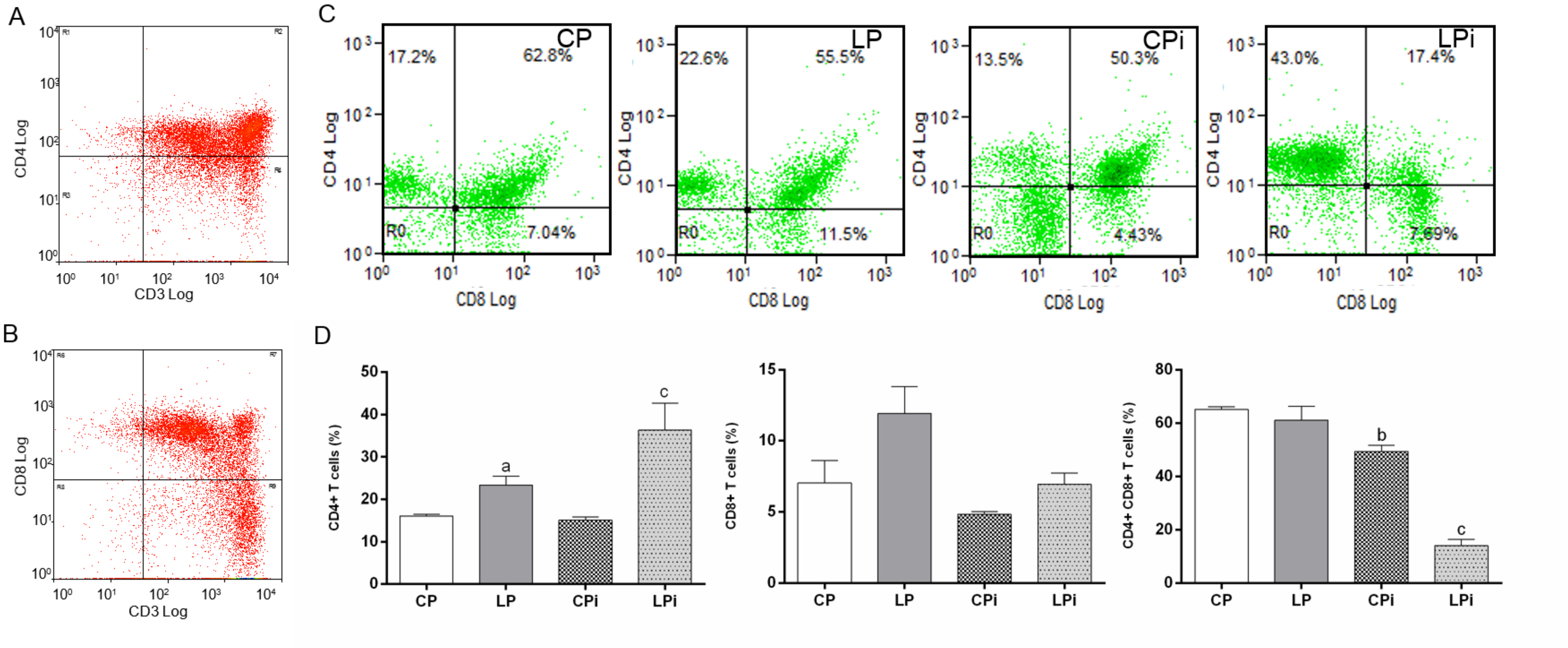

Supplementary Figure S2

Effect of protein malnutrition on liver and spleen weight in BALB/c mice infected with *L. infantum*. (A) Liver and **(B)** spleen weight gain at 14 dpi is expressed as a percentage of tissue/body weight in grams ± SEM (n=12). **(C)** Tissue weight data is expressed as a relation of organ (g) / body weight (g) ± SEM at 14 dpi. Two-way ANOVA analysis with Bonferroni *pos-hoc* test. Statistical differences due to diet: **a** ($p<0.001$), infection: **b** ($p<0.05$). CP: animals fed 14% protein diet; LP: animals fed 4% protein diet, CPi: animals fed 14% protein diet and infected; LPi: animals fed 4% protein diet and infected.

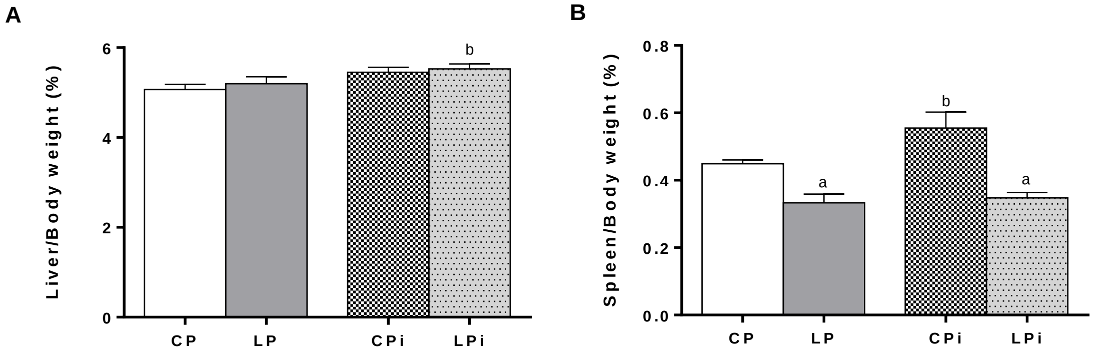

C

Group	Liver (g) / body weight (g) ± SEM	Spleen (g) / body weight (g) ± SEM
**CP**	0.0515 ± 0.0012	0.0045 ± 0.0001
**LP**	0.0520 ± 0.0015	0.0033 ± 0.0003[a]
**CPi**	0.0545 ± 0.0011	0.0056 ± 0.0005[b]
**LPi**	0.0553 ± 0.0011[b]	0.0035 ± 0.0002[a]

Supplementary Figure S3

mRNA levels of apoptotic genes in the thymus of protein malnourished BALB/c mice infected with *L. infantum*. *Bcl2, Survivin, Bax, Bid, Caspase3 and Apaf1* mRNA expression levels measured by qPCR in thymocytes of each experimental group. The values are expressed as normalized ratios between the target gene expression and the geometric median of the genes *Atp*-5, *Gapdh* and *Cyc*-1. CP: animals fed 14% protein diet; LP: animals fed 4% protein diet, CPi: animals fed 14% protein diet and infected; LPi: animals fed 4% protein diet and infected. Two-way ANOVA with Bonferroni *post-hoc* test. Statistical differences due to diet (**a,** p<0.05).

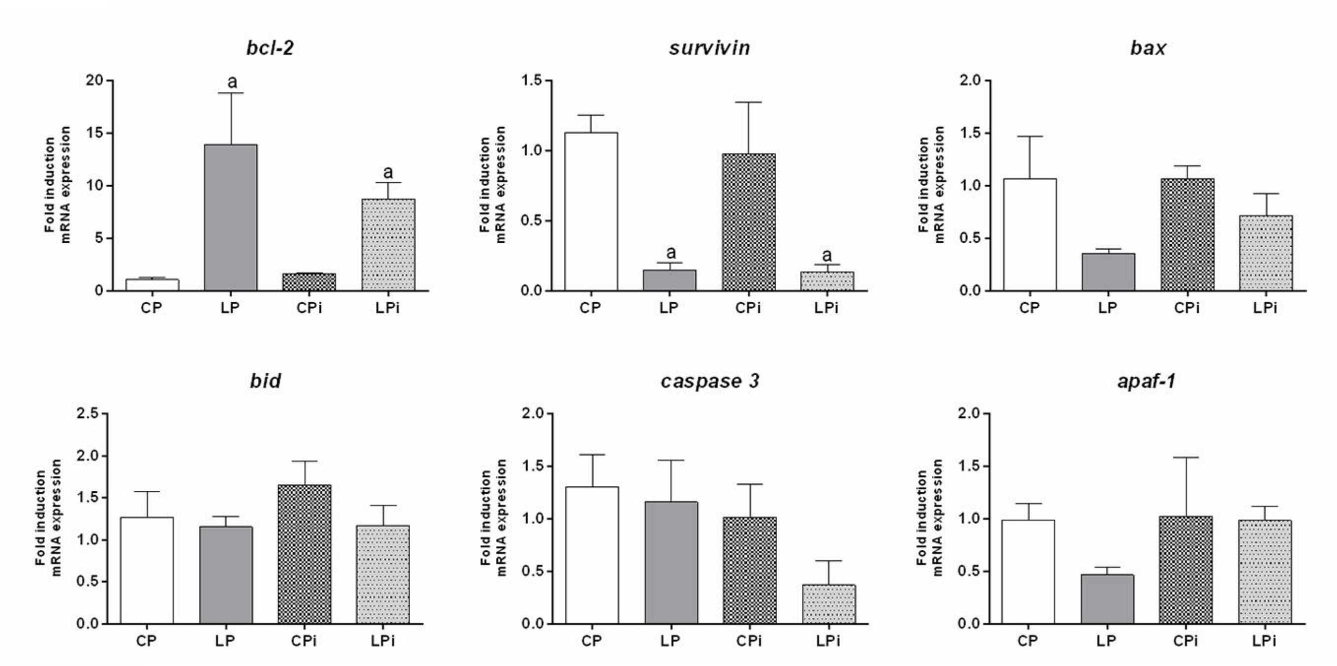

Supplementary Figure S4

CXCR3 expression on thymocytes subsets. CXCR3 expression om thymocytes subsets. The percentage of thymic CD4$^+$ **(A)**, CD8$^+$ **(B)** or CD4$^+$CD8$^+$ **(C)** T cells expressing the chemokine receptor CXCR3 was determined by flow cytometry. The values are expressed as average ± SEM. Left panels: Percentage of T cells expressing CXCR3 among groups. Right panels: Representative plots of T cell subpopulations expressing CXCR3 at 14 dpi. Upper panel: CD4$^+$; middle panel: CD8$^+$; lower panel: double positive T cells.

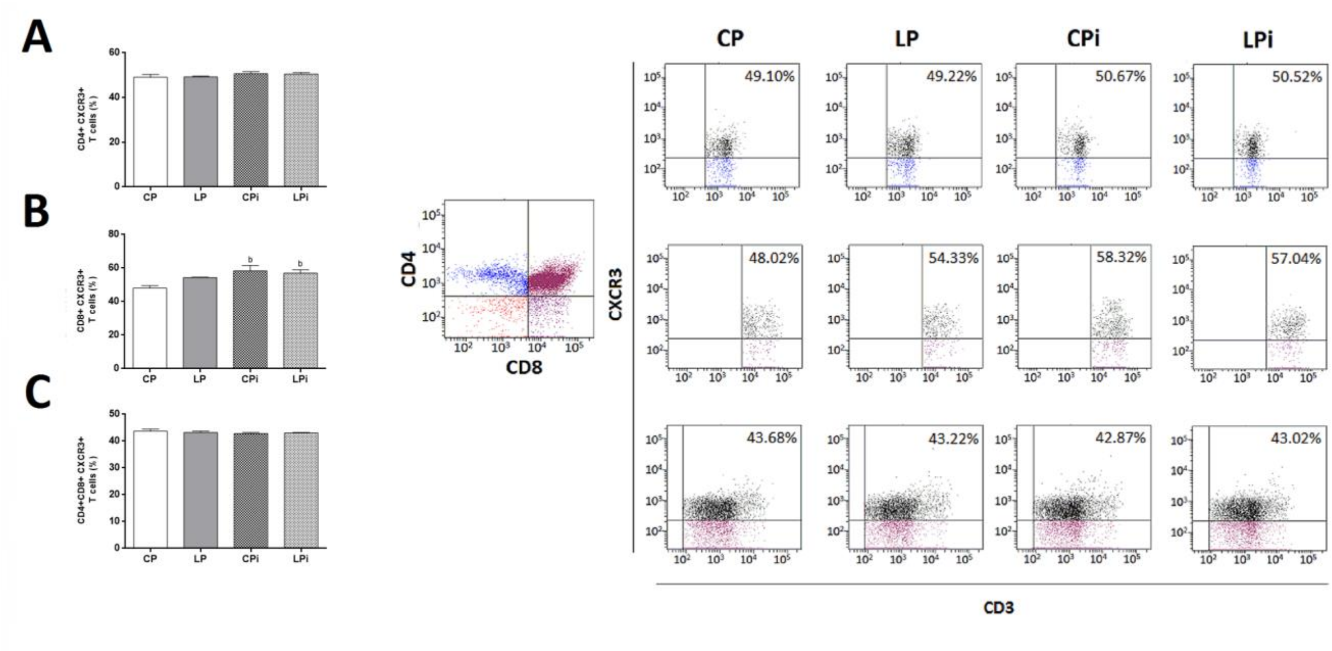

Supplementary Table S1

Effect of protein malnutrition on *L. infantum* infection in thymus cellularity of BALB/C mice

Treatment	Absolute thymocyte number ($x10^7$ cells)	Percentage of loss or gain respect to CP diet
CP	5.17 ± 0.48	-
LP	1.43 ± 0.35[a]	-72.4 ± 6.70
CPi	6.35 ± 0.15	+22.9 ± 2.84
LPi	1.05 ± 0.18[c]	-79.8 ± 3.42

CP: animals fed 14% protein diet; LP: animals fed 4% protein diet, CPi: animals fed 14% protein diet and infected; LPi: animals fed 4% protein diet and infected. Letters indicate statistical differences using a Two-way ANOVA analysis with Bonferroni *pos-hoc* test. Statistical differences due to diet: **a** ($p<0.0001$), and interaction between diet and infection: **c** ($p<0.05$).

Supplementary Table S2. Sequences of primers used for real time qPCR

Accession Number	Target	Forward Primer	Reverse Primer	Product size (bp)	Slope	Efficiency (E) (%)	Correlation coefficient (R^2)
NM_009741.4	***Bcl-2***	CTGAGTACCTGAACCGGCAT	AGTTCCACAAAGGCATCCCAG	70	3.19	102	0.99
NM_007527.3	***Bax***	TGCCTTGGACTGTGTCTT	AAAATGCCTTTCCCCTTCC	75	3.25	101	097
NM_007544.3	***Bid***	ATCCACAACATTGCCAGACA	TCCTTTTGTCTTCCTCCGAC	127	3.27	101	0.83
NM_001284409.1	***Casp3***	GAGCACTGGAATGTCATCTCG	AGGCCCATGAATGTCTCTCT	72	3.18	103	0.99
NM_001042558.1	***APAF1***	TGGGCTGCTTTCTTTCGATT	GATGTGTAACCAAGCCTTTGC	88	3.31	100	0.97
NM_009689.2	***Survivin***	ACAACCCGATAGAGGAGCATA	CATACAATTTTGTTCTTGGCTCT	133	3.16	103	0.99
NM_011337.2	***CCL3***	TTCCACGCCAATTCATCG	GCATTCAGTTCCAGGTCAG	151	3.39	98.5	0.95
NM_021704.3	***CXCL12***	GCTTCATCTGACTTCCGCTTCTC	CCACATCACTCTCCTCCCTTCC	77	3.33	99.8	0.97
NM_010512.4	***IGF1***	AAATCCCTTCCAACCAGTG	GATACAGAGACACCGATAGG	163	3.35	99.3	0.99
NM_009912.4	***CCR1***	GGCATCATCACCAGTATTATC	GCTTCAGGCTCTTGTAGG	133	3.39	98.7	0.98
NM_009917.5	***CCR5***	ATCCTGCCTCTACTTGTC	GCCTCTTCTTCTCATTCC	82	3.91	90.1	0.99
NM_009911.3	***CXCR4***	TCATCTACACTGTCAACCTCTAC	GGTGGCGTGGACAATAGC	86	3.47	97.1	0.99
NM_007722.4	***CXCR7***	GAGCACAGCATCAAGGAG	CCAACATACCAGGAAGACC	192	3.29	101	0.99
NM_010513.2	***IGF1R***	TTCGGACCAGTCTCGCCAAC	AAAGAGGAGCAAAGCCCAAATCG	121	3.13	100	0.99
NM_001301713.1	***CCR7***	ATGGACCCAGGTGTGCTTCT	CGACTCGTACAGGGTGTAGT	88	3.07	91	0.98
NM_001166625.1	***CCR9***	GGAGGCTGGTCTGCATTATC	CCAGGAATAAGGCTTGTGAGT	90	3.55	86	0.99
NM_001289726.1	***Gapdh***	GCCTTCCGTGTTCCTACC	CTTCACCACCTTCTTGATGTC	96	3.31	100	0.97
NM_025567.2	***Cyc1***	GGTGTCATTGCGAGAAGG	GGTGCCATCATCATACTCC	106	3.32	100	0.99
NM_016774.3	***ATPβ5***	TGAGTGTTGAGCAGGAGATTC	TTGGCGACATTGTTGATTAGC	148	3.39	97	0.99
AF285161.1	***UBC***	CTGTGAGAGCCGTGGATATTGG	GCACTTCCGTCTTTCAGCAAA	84	3.47	94	0.98
